# Ki-67 proliferation index to further stratify invasive breast cancer molecular subtypes: Northern African comparative cohort-study with external TCGA-BRCA and METABRIC validation

**DOI:** 10.11604/pamj.2022.41.170.31239

**Published:** 2022-03-02

**Authors:** Laila Akhouayri, Meriem Regragui, Samira Benayad, Nisrine Bennani Guebessi, Farida Marnissi, Giovanna Chiorino, Mehdi Karkouri

**Affiliations:** 1 Department of Biomedical Sciences, Genetics and Molecular Biology Laboratory, Faculty of Medicine and Pharmacy, Hassan II-Casablanca University, Rue Tariq Ibn Ziad, Casablanca, Morocco,; 2 Genomics Lab, Fondazione Edo ed Elvo Tempia, via Malta, Biella, Italy,; 3 Dipartimento di Scienze Cliniche e Biologiche, Università Degli Studi Di Torino, Via Giuseppe Verdi, Torino, Italy,; 4 Department of Pathology, Ibn Rochd University Hospital, Rue des Hôpitaux, Casablanca, Morocco

**Keywords:** Ki-67, estrogen, progesterone, immunohistochemistry, clustering, prognosis

## Abstract

**Introduction:**

breast cancer (BC) is a malignancy with very high incidence and mortality in Africa, especially in Western Africa, where more than 25 thousand deaths are registered every year. Not all BC have the same prognosis, and being able to personalize treatment and predict aggressiveness is of crucial importance. The purpose of our study is to explore further subdivisions associated with prognosis, beyond breast cancer molecular classification that is routinely established in pathology departments.

**Methods:**

we conducted a 5-year retrospective cohort study on 1266 invasive BC of Moroccan patients, collected at the Pathology Department of Ibn-Rochd University Hospital in Casablanca, and followed at King Mohammed VI National Centre for the Treatment of Cancers. We elaborated an Estimation-Maximization Clustering, based on the main BC biomarkers: Ki-67, HER2, estrogen and progesterone receptors, evaluated by immunohistochemistry. Two independent datasets (TCGA-BRCA and Metabric) were also analyzed to assess the external reproducibility of the results.

**Results:**

each molecular subgroup could be partitioned into two further subdivisions: Cluster1, with average Ki-67 of 16.26% (±11.9) across all molecular subgroups and higher frequency within luminal BC, and Cluster2, with average Ki-67 of 68.8%(±18) across all molecular subgroups and higher frequency in HER2 as well as in triple-negative BC. Overall survival of the two Clusters was significantly different, with 5-year rates of 52 and 37 months for Custer1 and Cluster2, respectively (p=0.000001). Moreover, mortality rates within the same molecular subgroup, especially in luminal B HER2-, varied remarkably depending on Cluster membership (6% for C1 and 18% for C2 after 1 year of follow-up). Two different algorithms to evaluate the prognostic importance, variable selection using random forests (VSURF) and Minimal depth, ranked the subdivision proposed as one of the 4 most influential features being able to predict patient survival better than several histoprognostic features, both in the Moroccan and in the external datasets.

**Conclusion:**

our results highlight a new refinement of the BC molecular classification and provide a simple and improved way to classify tumors that could be applied in low to middle-income countries. This is the first study of its kind addressed in an African context.

## Introduction

Globally, BC is the most common cancer in women, with approximately 2.2 million new cases diagnosed in 2020 (11.7% of all cancers, both sexes, all ages included). Its incidence rate varies significantly between regions of the World, with its peak in Asia followed by the Central, Eastern, and Western parts of Europe, North and Latin America and Africa [1]. Mortality within the African continent ranges from 5090 events in Southern Africa as the least concerned region, to 25626 in Western Africa, making it the most concerned region of Africa by this type of cancer [1]. In 2020, 11747 new BC cases were recorded in Morocco, representing 19.8% of all cancers in women and the first diagnosed cancer. It is the first also in terms of mortality (3695 estimated deaths) and prevalence (31420 cases for 5-years prevalence) [2]. The cancer registry of the greater Casablanca Region (2016-2020), according to the latest report developed by the Department of Epidemiology and Disease Control of the Ministry of Health, estimates the frequency of BC at 35.8%, with a peak recorded between 55 and 59 years [3]. Currently, analysis of tumor morphology and immunohistochemistry (IHC) play a central role in the evaluation of invasive BC. The most commonly used immunohistochemical prognostic and therapeutic biomarkers include estrogen (ER) and progesterone receptors (PgR), human epidermal growth factor receptor 2 (HER2), and proliferation index (Ki-67) which translates the proliferative and aggressive potential of the tumor. Moreover, the majority of the 2013 St. Gallen panel of experts agreed on the use of these biomarkers as surrogates for the definition of BC molecular subgroups [4]. Unlike genomic profiling, which can be complex and much more expensive for routine clinical use, IHC is accessible to the majority of pathology laboratories, especially in low-income countries like Morocco, and easy to use. Moreover, the routinely established BC molecular subgroups still display internal heterogeneity in terms of prognosis [5]. In this study, we explore if IHC biomarkers can be used to better discriminate prognostic groups within the framework of the molecular classification of invasive BC, by applying statistical partitioning methods to find new subdivisions. We test their association with survival using both a Moroccan dataset from our institution and two external independent datasets.

## Methods

**Study design and setting:** this is a comparative retrospective cohort-study including Moroccan BC patients with 5 years of follow-up, the TCGA-BRCA and Metabric datasets with 13 and 30 years of follow-up, respectively. All invasive breast carcinomas recorded in the databases were included. In contrast, benign tumors or tumors of uncertain malignancy; tumor recurrences; breast cancer in men; patients with incomplete/equivocal immunohistochemical status were all discarded.

**Study population:** general and clinical data on 1266 Moroccan patients with invasive BC, recorded from January 1^st^, 2013 to March 30^th^, 2018, were initially collected at the Pathology Department of Ibn Rochd University Hospital of Casablanca, and 165 of them were followed at King Mohammed VI Center for the Treatment of Cancers, Casablanca, where their 5 year-follow-up survival data were collected.

**Data collection:** determination of ER, PgR, Ki-67 and HER2 status on the Moroccan dataset was performed by immunohistochemical staining on sections from formalin-fixed paraffin-embedded (FFPE) tissues obtained either from pre-surgical core biopsies or surgical specimens.

**Immunohistochemistry:** the tissues were cut to 5um, then deparaffinized in toluene. This step was followed by rehydration by immersing the slides in decreasing alcohol baths and distilled water. The sections underwent heat induced epitome retrieval and the peroxidase blocking. Application of the primary antibody was then performed by using the Herceptest kit (DAKO) for HER2, the IR657 monoclonal mouse anti-human Estrogen receptor antibody for ER, the IR068 flex monoclonal mouse anti-human progesterone receptor for PgR and the IR626 flex monoclonal mouse anti-human anti-Ki-67 antigen for Ki-67, while viewing was done using the Dako Envision™ detection kit. Immunohistochemistry was performed using the PT link controller and the autostainer link controller. Afterwards, each section was treated with 100µl of Dako Envision Flex/HRP visualization reagent coupled to the secondary antibody. The slides were then examined under a Leica DM1000 optical microscope. The pathological evaluation and interpretation of the staining was carried out by the same team of four pathologists at the Pathology Department of Ibn Rochd University Hospital, and the final scoring was given by a consensus, leading to consistent pathological reporting. The histological subtyping and Scarff-Bloom-Richardson (SBR) grading were assessed in concordance with standard guidelines. Scoring is based on the ASCO/CAP recommendations for estrogen receptor (ER) and PgR [6] considering any nuclear staining in at least 1% of invasive tumour cells as positive and according to 2018 ASCOP/CAP recommendations for HER2 [7]. As for Ki-67, the cut-off was set at 20%, one of the easiest levels of staining to characterize [8]. The results for ER, PgR and Ki-67 were recorded therefore as the percentage of immunoreactive cells over up to 2000 neoplastic cells. Two external datasets were retrieved from public repositories.

**TCGA-BRCA dataset:** retrieved from genomic data commons (GDC) Data Portal of the National Cancer Institute [9] and initially composed of 963 invasive BC records. The dataset contains the following features: ESR1, PGR, ERBB2 and MKI67 (genes expression Z-scores), lymph nodes stage, neoplasm disease stage, menopause status, tumor stage, altered genome fraction, metastatic stage, cancer type, sample initial weight, mutation count, micrometastasis detection, ethnicity category, histologic type, race category, lymph node ratio (LNR), overall survival status, overall survival months (over 13 years of follow up), disease free months, disease free status, diagnosis stage, positive lymph node count, lymph node examined number. After missing values filtering, the final number of remaining BC records was 624. Twenty-four (24) patients were recorded as Hispanic or Latino; 458 as non-Hispanic or Latino and the status was missing for 143 patients. As their race category: 428, 70, 38, 1 were recorded as white, black, or African American, Asian, American Indian, or Alaska Native respectively and the variable was missing for 88 patients.

**Metabric dataset:** it contains 1885 BC records and was retrieved initially fromthe cBioPortal [10]. It contains the following histoprognostic features: MKI67 (genes expression Z-score); ER/PgR/HER2 (over expressed/under expressed); age at diagnosis (years); cancer type; cellularity; chemotherapy; neoplasm histologic grade; HER2 status measured by SNP6; histologic subtype; hormone therapy; inferred menopausal state; primary tumor laterality; nottingham prognostic index; radio therapy; tumor size; patients vital status; lymph nodes examined positive; mutation count; overall survival time (over 30 years of follow up); survival status (censored/dead). There were no missing values in this dataset. No social or demographic feature was present in this dataset. Briefly, the following datasets were used: i) the TCGA-BRCA dataset, containing 624 records with complete information on ESR1, PGR, ERBB2 and MKI67 (gene expression Z-scores) and follow-up; ii) the Metabric dataset, with complete information on MKI67 (gene expression Z-score), ER/PgR/HER2 (over expressed/under expressed) and follow-up on 1885 records.

**Definitions:** for all the datasets, Ki-67, ER, PgR and HER2 variables were extracted for further analysis. Subsequently, BC was systematically classified into five intrinsic subgroups, as follows: i) LuminaA (LumA): ER+ and/or PgR+; HER2-; low Ki-67; ii) luminal B HER2+ (LumB HER2+): ER+ and/or PgR+; HER2+; high Ki-67; iii) luminal B HER2- (LumB HER2): ER+ and/or PgR+; HER2-; high Ki-67; iv) pure HER2: ER- and PgR-; HER2+; irrespective of Ki-67; v) triple negatives (TN): ER- and PgR-; HER2-; irrespective of Ki-67. As for Metabric and TCGA-BRCA dataset which contain Z-scores, all the samples with positive Z-scores were considered with high expression of MKI67, ER, PgR and HER2 and vice-versa.

**Statistical analysis:** estimation-maximization (EM) clustering was assessed on the Moroccan and Metabric datasets because it is more suitable to the nature of their attributes. In contrast, partition around medoids (PAM), hierarchical and K-means clustering were applied to partition TCGA-BRCA dataset. For all the three datasets, a minority of records with equivocal HER2 status (HER2 2+) that couldn´t be assessed by fluorescence in situ hybridization (FISH) and ER-PgR+ records were discarded because they represented a minority of the study population and there is much debate about the existence of this combination. The optimal number of k-clusters was calculated by validation measures using the “clValid package” in R, that helps to simultaneously select multiple clustering algorithms, validation metrics, and cluster counts in a single function call, in order to determine the most suitable method and optimal number of clusters. Cluster membership prediction was assessed by Rapidminer studio software v9.9, which splits the dataset in a training set with 70% of the samples and a test set with the remaining samples before running the prediction models. Eight prediction algorithms were applied: naive bayes (NB), generalized linear model (GLM), fast large margin (FLM), deep learning (DL), decision tree (DT), random forest (RF), gradient boosted trees (GBT) and support vector machine (SVM). The used predictor variables were: ER, PgR, HER2 and Ki-67. To explore which model showed the strongest predictive ability, the latter were evaluated by ten metrics: accuracy, area under the Curve Receiver Operating Characteristic (ROC-AUC), precision, recall, F-measure, sensitivity, specificity, classification error and total running time. The cross-validation option was used, which helps assess the model's ability to classify new data, to avoid problems like overfitting, and estimates how accurately this predictive model performs. Important variables selection and their influence on the survival prediction of BC patients were assessed by random forest Src package, using two attributes selection algorithms: Vimp and the minimal depth. Surviving patients were censored at the date of last follow-up; survival plots according to BC molecular subgroups were drawn using the Kaplan-Meier method to evaluate their overall survival (OS) rates. The log-rank test was applied to assess the survival difference between strata.

**Ethical considerations:** the study was approved by the local ethical committee.

## Results

**Figure 1:**
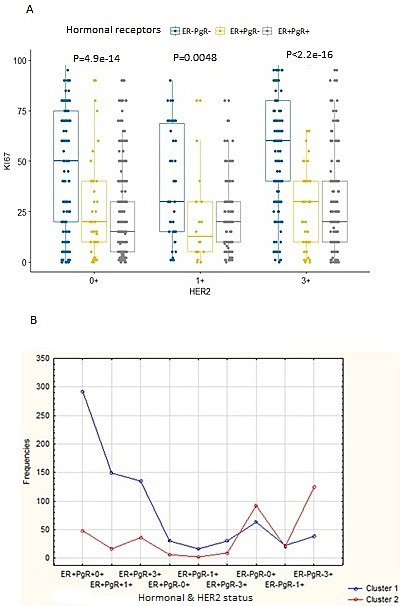
estimation maximization clustering outcome on the Moroccan dataset

**Figure 2:**
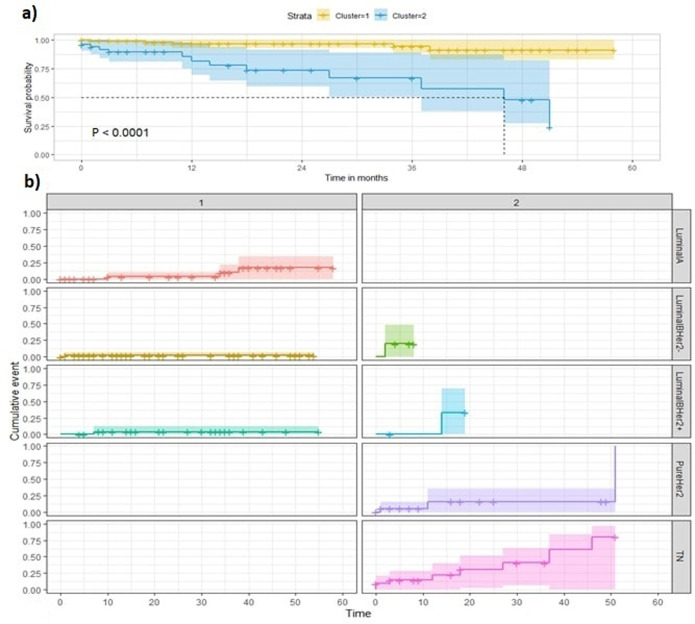
five (5) years overall survival analysis for Moroccan dataset patients

**Moroccan dataset:** after exclusion of equivocal HER2 2+ and of ER-PgR+ cases, the remaining 1128 patients were stratified in 9 phenotypes {ER+PgR+HER2 0+; ER+PgR+HER2 1+; ER+PgR+HER2 3+; ER+PgR-HER2 0+; ER+PgR-HER2 1+; ER+ PgR-HER2 3+; ER-PgR-HER2 0+; ER-PgR- HER2 1+; ER- PgR- HER2 3+} (Figure 1). Estimation-maximization clustering was applied on all the 9 phenotypes to test the existence of intra-subgroup heterogeneity (Figure 1), and the “v-fold cross-validation” was used to automatically determine the appropriate number of clusters. Each phenotype was statistically divided into two further subdivisions, as follows: C1 (cluster1), including patients with low Ki-67 (16.26±11.9 as mean percentage across all molecular subgroups) and C2 (Cluster2), including patients with high Ki-67 (68.8±18 as mean percentage across all molecular subgroups). As long as ER was expressed, C1 was statistically found to be the most frequent. However, C2 was frequent in ER-HER2+phenotypes, (Table 1). The percentage of C2 patients was higher in the HER2 (76.5%) and the triple negative (57%) molecular subgroups, thus reflecting their high mitotic activity and worst prognosis. In contrast, C1 was the most frequent in luminal A/luminal B HER2- and luminal B HER2+ subgroups (87% and 78%, respectively), which reflects their favorable prognosis. Correspondingly, the 4 biomarkers ER, PgR, Ki-67 and HER2 showed a stable and accurate potential to predict BC patients cluster´ membership according to several prediction algorithms (Table 2). Survival analysis according to C1 and C2 belonging showed a difference in mean survival (log-rank test p=0.000001) which was approximately 52 months and 37 months respectively, suggesting a poorer survival for the latter (Figure 2). The computation of “cluster membership” combined to “molecular subgroups” is reported in (Figure 2) where the left column refers to Cluster1 membership; contrary to the right column that refers to Cluster2 membership.

**Table 1:** Ki-67 distributions in cluster 1 and cluster 2 according to ER/PgR/HER2 status

ER-PgR-	HER2 0+ C1	HER2 0+ C2	HER2 1+ C1	HER2 1+ C2	HER2 3+ C1	HER2+ 3+ C2
Patients (%)	40.6	59.3	52.3	47.6	23.4	76.5
Minimal-maximal values	0-40	45-100	1-50	60-90	0-70	75-100
Mean Ki-67	16.4	73	20.4	71.7	42.3	92.5
Standard deviation	13	15.4	16.3	8.2	21.4	9.2
Total	155		42		161	
**Molecular subgroup**	**Triple negative**	**Triple negative**	**Triple negative**	**Triple negative**	**Pure HER2**	**Pure HER2**
ER+PgR-	HER2 0+ C1	HER2 0+ C2	HER2 1+ C1	HER2 1+ C2	HER2 3+ C1	HER2 3+ C2
Patients (%)	83.3%	16.6%	88.8%	11.1%	77	23
Minimal-maximal values	0-40	50-90	0-40	60-80	0-65	100-100
Mean Ki-67	16.86	68.33	14.7	70	26.3	100
Standard deviation	13.32	15.7	13.7	14	20.2	0
Total	36		18		39	
**Molecular subgroup**	**LuminalA/B HER2-**	**LuminalA/B HER2-**	**LuminalA/B HER2-**	**LuminalA/B HER2-**	**Luminal B HER2+**	**Luminal B HER2+**
ER+PgR+	HER 2 0+ C1	HER2 0+ C2	HER2 1+ C1	HER2 1+ C2	HER2 3+ C1	HER2 3+ C2
EM clusters	C1	C2	C1	C2	C1	C2
Patients (%)	85.8	14.1	90.3	9.7	79	21
Minimal-maximal values	0-20	25-90	0-30	40-100	0-40	45-100
Mean Ki-67	9.5	45	14.8	51.7	17.5	71.1
Standard deviation	6.3	18	9.1	15.1	12.5	16.4
Total	340		165		171	
Molecular subgroup	Luminal A/B HER2-	Luminal A/B HER2-	Luminal A/B HER2-	Luminal A/B HER2-	Luminal B HER2+	Luminal B HER2+

ER: estrogen receptor; PgR: progesteron receptor; HER2: human epidermal growth factor receptor 2; EM: estimation-maximisation; C1: cluster1; C2: cluster2 (For convenience and clarity of the clutter-free table, the luminal A HER2- subgroup has been combined in the same cell as the Luminal B HER2- subgroup because they both form the luminal type)

**Table 2:** evaluation summary of eight prediction algorithms for clusters membership prediction

Prediction algorithms	Accuracy (%)	AUC (%)	Precision (%)	Recall (%)	F- measure (%)	Sensitivity (%)	Specificity (%)	Classification error (%)	Scoring time (ms)
NB	80±2.4	80	81.4	91.9	86.2	91.9	54.1	20	190
GLM	80.8±2.2	83.1	79.2	97.7	87.4	97.7	44.9	19.2	246
FLM	80.5±3.4	78.4	85.3	86.4	85.8	86.4	68.1	19.5	159
DL	79±1.7	78.6	78.4	96.0	86.2	96	41.8	21	429
RF	80±4.3	80.3	82.9	89.1	85.9	89.1	60.4	20	657
GBT	81.4±2.5	81.1	82.7	92.4	87.2	92.4	57.5	18.6	1000
SVM	68.4±0.7	52.5	68.4	100.0	81.2	100	0	31.6	2000
DT	81.4±3	81.6	80.9	95.5	87.6	95.5	51.1	18.6	166

Rows: prediction algorithms; NB: naive bayes; GLM: generalised linear model; FLM: fast large margin; DL: deep learning; DT: decision tree; RF: random forest; GBT: gradient boosted trees; SVM: support vector machine; evaluation metrics (columns): accuracy, area under the curve receiver operating characteristic (ROC-AUC); precision, recall, F-measure, sensitivity, specificity, classification error and total running time

**Figure 3:**
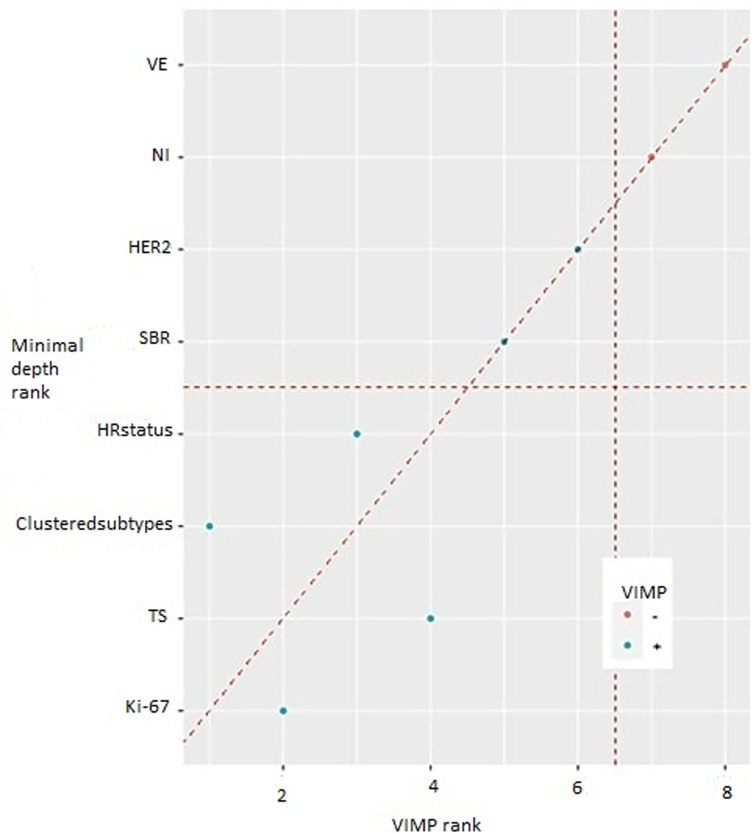
comparing minimal depth and vimp rankings

It is also to be mentioned that within these 165 women with follow-up information no BC record was found simultaneously belonging to luminal A subgroup and Cluster2. Conversely, no BC record belonging to the HER2 and TN subgroups was clustered as Cluster1. Hence the non-assessment of their respective curves. The latter led to a higher cumulative incidence in C2 than in C1, especially in the TN (81%). Mortality rates also differed between C1 and C2 within luminal B/HER2+ (6% and 36%) and within luminal B/HER2- tumors (6% and 18.5%), respectively (Figure 2). The stratification of patients by Ki-67 only gave a p-value of 0.000153, higher than the p-value given by cluster membership stratification (p=0.000001), or by cluster membership and molecular subgroup membership stratification (p=0.000021). Consequently, the new variable called “clustered subtypes” referring to cluster and molecular subgroup membership was voted by both feature selection algorithms as one of the most influential features on BC patient survival, together with Ki-67, tumor size and hormonal receptors status (Figure 3). Points on the red dashed line are ranked equivalently, points above have higher vimp ranking indicating the variables are more sensitive to misspecification; those below have higher minimal depth ranking, indicating they are better at dividing large portions of the population. The farther the points are from the line, the more the discrepancy between measures. The figure presents all the variables: vascular emboli (VE); nodes involvement (NI); hormonal receptor status (HR status); tumor size (TS). ER and PgR are initially continuous variables. For more clarity, we decided to combine them in the same variable as they both refer to hormone receptors “HR”. The variable was categorized as follows: ER ≥1% et PgR ≥1%=HR positive; ER ≥1% and PgR<1% = HR positive; ER<1% and PgR <1% = HR negative.

### Comparative analysis using external datasets

**Optimal number of k-clusters:** in the TCGA-BRCA dataset, hierarchical clustering outperformed the other algorithms under each internal measure, with 2 as the optimal number of clusters. In terms of the stability measures, average proportion of non-overlap (APN) and average distance between means (ADM) evaluated PAM clustering with 2 clusters as the most stable. Accordingly, five out of seven measures voted for the “2-clusters partition” (Table 3). In terms of the Metabric dataset, the optimal number of clusters was assessed using EM analysis because it is more suitable to its attributes. Each molecular subgroup was found to be statistically divided into C1, including patients with a low Ki-67 (-0.15±0.8 as mean Z-score) and C2, including patients with a high Ki-67 (2.4±0.5 as mean Z-score). Their dispersion showed that C1 membership greatly decreases when HER2 is over expressed. In reverse, the latter increased to reach its peak in HER2-ER+PgR+ phenotype, and the distributions within C1 and C2 were significantly different (p =0.000012). All these results converge with those obtained in the Moroccan dataset, confirming that C1 has a lower average Ki-67 index and is mainly associated with hormone dependent tumors (luminal subgroups). In contrast, C2 has higher average Ki-67 and is more associated with hormone independent tumors.

**Table 3:** summary of clustering methods and internal/stability measures for optimal number of clusters calculation in TCGA-BRCA dataset

Clustering method	Internal measures	2 clusters	3 clusters	4 clusters	5 clusters
Hierarchical	Connectivity	2.9290	10.4544	24.9115	28.7694
	Dunn	0.2603	0.0933	0.0915	0.0915
	Silhouette	0.463	0.2450	0.2619	0.2328
K-means	Connectivity	62.1687	152.5885	133.0683	149.8202
	Dunn	0.0349	0.0183	0.0315	0.0335
	Silhouette	0.4114	0.2430	0.3040	0.3036
PAM	Connectivity	63.6940	69.7341	161.0004	194.0429
	Dunn	0.0437	0.0418	0.0214	0.0328
	Silhouette	0.4163	0.4331	0.2987	0.2888
	Stability measures	APN	AD	ADM	FOM
	Score	0.0808	1.6819	0.3595	0.8451
	Clustering method	PAM	K-means	PAM	K-means
	Optimal number of clusters	2	5	2	5

PAM: partition around medoids; APN: average proportion of non-overlap; AD: average distance; ADM: average distance between means; FOM: figure of merit

**Overall survival analysis depending on clusters membership:** overall survival (OS) Kaplan-Meier analysis on TCGA-BRCA, based on cluster membership, shows two distinct curves (p=0.00042). Cluster2 of the luminal B/HER2- subgroup has a much more defavorable survival probability (30%) than that of C1 (87.5 %) after 50 months of follow-up. This suggests that prognosis could be better refined if patients routinely classified in the same molecular subgroup are further subdivided in sub clusters. In terms of the Metabric records, the mean survival of C1 and C2 was 14 and 12.8 years, respectively. The 30-year OS rates based on cluster membership within each molecular subgroup showed that patients belonging to C1 of TN subgroup have approximately the same survival outcome as those belonging to C1 of luminal B/HER2+ which is 50% survival probability after 15 years of follow-up, in contrast to C2 of TN which reaches the same survival probability after 13.5 years of follow up only. In conclusion, the partition of survival curves depending on molecular subgroups and cluster membership is more precise and significant than just the molecular classification partition.

**Clustered subtypes; variable importance:** in the TCGA-BRCA dataset, menopause status, tumor stage, fraction genome altered and “clustered subtypes” were the most important explanatory features in predicting patient survival by VIMP. The minimal depth method, oppositely, indicated eight variables with a higher impact. Both methods highlight the importance of tumor stage and “clustered subtypes” in predicting survival probability. As for the Metabric dataset, the “clustered subtypes” variable was classified by both Vimp and minimal depth methods as the fourth most influential on survival, within all the 22 predictive features analyzed.

## Discussion

This study explores the possibility of partitioning BC molecular subgroups in order to better define patient survival. The partitioning approach was applied starting from 1128 Moroccan BC records, and then tested on two external independent datasets, also used to further validate the clinical significance of the newfound subdivisions, in terms of survival prediction. We found that the routinely established BC molecular classification could be further refined by only using Ki-67, ER, PgR and HER2 variables. Each subtype can be subdivided in two distinct clusters with significantly different Ki-67 distribution and survival outcomes. Tumors belonging to the cluster with low mitotic activity (C1) are overrepresented in the luminal subtype, while HER2 and TN subtypes are enriched in tumors belonging to the cluster with high mitotic activity (C2). This partitioning is also associated with overall survival (OS) and is equally or even more important than tumor size in predicting outcome. Indeed, Marwah *et al*. [9] revealed that a higher Ki-67 was found with a size greater than 5 cm while tumors smaller than 2cm showed a lower rate. This finding was also confirmed by Querzoli *et al*. [10]. Similarly, several studies showed a positive correlation between Ki-67 and the tumor histological grade. However, we can point out that our analysis on the Moroccan dataset did not rank histological grade, unlike cluster membership and tumor size, among the most important variables able to predict survival.

Another interesting result is the presence of C1 samples within TN tumors. Histological subtype could in part explain this result, with cystic adenoid carcinoma as a typical example. Although it does not express hormone receptors and does not over express HER2, it shows a low proliferation index [11,12]. Ki-67 is also reported to be higher in triple negative breast cancer (TNBC) of no special type compared to TNBC of other histological subtypes [13]. It has been suggested by some authors as a prognostic and predictive marker for TNBC [14,15]. Keam's study proposed a 10% Ki-67 cut-off to define two different prognostic subgroups: the first one with high Ki-67, which despite a better response to chemotherapy was more aggressive, and the second one with low Ki-67, which showed a lower aggressiveness but also a lower response to chemotherapy [16]. Our results confirm these findings, with TNBCs partitioned into 2 further subdivisions: C1 and C2, with mean Ki-67 of 16.4±13% and 73±15.4%, respectively. On the other hand, Bartlett *et al*. recently reported that, despite little concordance at the single tumor level, heterogeneity within ER+ tumors in terms of prognosis is detectable and confirmed by different BC multiparameter tests [17]. In addition, Aleskandarany *et al*. confirmed that within the luminal B/HER2- subgroup, the group with high proliferation index had worse evolution and prognosis than the group with low Ki-67. This supports our results, with some cases within the luminal B/HER2- molecular subgroup clustered in C2 and showing worse survival in all the three analyzed datasets. Therefore, BC molecular subgroups should be considered as a spectrum of diseases [18].

In terms of survival, we showed that subtype partitioning helps to refine the prognosis. Kyung Lee *et al*. demonstrated that the combination of p53 and Ki-67 has the best predictive power, especially for long-term OS in the luminal A subgroup [19]. Interestingly, in our study we were able to highlight that not only luminal A but also the other molecular subtypes, luminal B in particular, could benefit from a further refinement based on Ki-67. It should be noted that luminal tumors would represent a genotypically heterogeneous group with some tumors exhibiting chromosomal instability with aneuploidy and others without genomic instability and diploids [20]. On the other hand, no significant prognostic difference could be established for HER2 and TN tumors, since for our Moroccan cohort survival information was complete only for a small subset of patients. Therefore, we repeated the analyses on TCGA-BRCA and Metabric, which allowed us not only to extend and validate our clustering results to a broader context but also to confirm the intrinsic subdivisions proposed within the molecular subgroups have a clinical prognostic impact. TCGA-BRCA and Metabric contain a very wide panoply of prognostic features and cover even genomic data for each patient, therefore they could be further exploited to identify additional biomarkers translatable to the clinics.

## Conclusion

Our results suggest that Ki-67 allows a better definition of BC prognosis within each molecular subtype. To our knowledge, this is the first study addressing an African population and relying on routine immunohistochemical techniques only. This makes our proposed stratification affordable in all pathology departments, unlike gene expression profiling which is neither widely available nor accessible, especially in low-income countries.

### What is known about this topic

- Ki-67 is a marker that helps refining the breast carcinomas molecular subgroups according to their proliferative potential;
- Five (5) molecular subgroups can be assessed: luminal A, luminal B HER2+, luminal B HER2-, triple negatives and pure HER2.

### What this study adds

- Routinely established BC molecular classification can be furthermore refined; each molecular subgroup is found to be significantly sub divided in two other sub divisions that converge with a specific prognosis and survival outcome;
- HER2 and triple negative tumors mainly fall in sub divisions with high mitotic activity reflected by a high KI-67 mean; while luminal A/B tumors are more likely to belong to low mitotic activity subdivisions reflected with a low KI-67 mean;
- The overall survival of BC patients belonging to the same molecular subgroup but different clusters is significantly different, especially for Luminal B HER 2+ and Luminal B HER2- subgroups.
